# Urine-based multi-omic comparative analysis of COVID-19 and bacterial sepsis-induced ARDS

**DOI:** 10.1186/s10020-023-00609-6

**Published:** 2023-01-26

**Authors:** Richa Batra, Rie Uni, Oleh M. Akchurin, Sergio Alvarez-Mulett, Luis G. Gómez-Escobar, Edwin Patino, Katherine L. Hoffman, Will Simmons, William Whalen, Kelsey Chetnik, Mustafa Buyukozkan, Elisa Benedetti, Karsten Suhre, Edward Schenck, Soo Jung Cho, Augustine M. K. Choi, Frank Schmidt, Mary E. Choi, Jan Krumsiek

**Affiliations:** 1 Department of Physiology and Biophysics, Institute for Computational Biomedicine, Englander Institute for Precision Medicine, Weill Cornell Medicine, New York, NY 10021 USA; 2 Division of Nephrology and Hypertension, Joan and Sanford I. Weill Department of Medicine, New York, NY USA; 3 Division of Pediatric Nephrology, Department of Pediatrics, Weill Cornell Medicine, New York, NY USA; 4 New York-Presbyterian Hospital, New York, NY USA; 5 Division of Pulmonary and Critical Care Medicine, Department of Medicine, Weill Cornell Medicine, New York, NY USA; 6 Division of Biostatistics, Department of Population Health Sciences, Weill Cornell Medicine, New York, NY USA; 7 Bioinformatics Core, Weill Cornell Medicine –Qatar, Qatar Foundation, Doha, Qatar; 8 Proteomics Core, Weill Cornell Medicine –Qatar, Qatar Foundation, Doha, Qatar

**Keywords:** COVID-19, Acute respiratory distress syndrome (ARDS), Multi-omic, Mortality signature, Network-based, Computational analysis, Metabolomics, Proteomics, Mitochondrial dysfunction

## Abstract

**Background:**

Acute respiratory distress syndrome (ARDS), a life-threatening condition during critical illness, is a common complication of COVID-19. It can originate from various disease etiologies, including severe infections, major injury, or inhalation of irritants. ARDS poses substantial clinical challenges due to a lack of etiology-specific therapies, multisystem involvement, and heterogeneous, poor patient outcomes. A molecular comparison of ARDS groups holds the potential to reveal common and distinct mechanisms underlying ARDS pathogenesis.

**Methods:**

We performed a comparative analysis of urine-based metabolomics and proteomics profiles from COVID-19 ARDS patients (n = 42) and bacterial sepsis-induced ARDS patients (n = 17). To this end, we used two different approaches, first we compared the molecular omics profiles between ARDS groups, and second, we correlated clinical manifestations within each group with the omics profiles.

**Results:**

The comparison of the two ARDS etiologies identified 150 metabolites and 70 proteins that were differentially abundant between the two groups. Based on these findings, we interrogated the interplay of cell adhesion/extracellular matrix molecules, inflammation, and mitochondrial dysfunction in ARDS pathogenesis through a multi-omic network approach. Moreover, we identified a proteomic signature associated with mortality in COVID-19 ARDS patients, which contained several proteins that had previously been implicated in clinical manifestations frequently linked with ARDS pathogenesis.

**Conclusion:**

In summary, our results provide evidence for significant molecular differences in ARDS patients from different etiologies and a potential synergy of extracellular matrix molecules, inflammation, and mitochondrial dysfunction in ARDS pathogenesis. The proteomic mortality signature should be further investigated in future studies to develop prediction models for COVID-19 patient outcomes.

**Supplementary Information:**

The online version contains supplementary material available at 10.1186/s10020-023-00609-6.

## Background

The ongoing SARS-CoV-2 induced coronavirus disease 2019 (COVID-19) pandemic has been a major impediment to human life globally (UNDP 2021; Chriscaden 2020). One of the main complications of severe COVID-19 is acute respiratory distress syndrome (ARDS). ARDS is a common presentation of critical illnesses, including severe infections, major injury, or inhalation of irritants (Han and Mallampalli 2015). While COVID-19-related ARDS and ARDS originating from other pathologies (hereby referred to as non-COVID-19 ARDS) have overlapping clinical features, COVID-19 ARDS is characterized by a protracted hyperinflammatory state and higher rates of thrombosis (Grant et al. 2021; Helms et al. 2020; Hue et al. 2020; Bain et al. 2021; Brault et al. 2020; Dostálová and Dostál 2019; Robinson and Krasnodembskaya 2020; Levitt and Rogers 2016; Overmyer et al. 2021; Shen et al. 2020). The field currently lacks etiology-specific therapies and reliable predictors of heterogeneous patient outcomes (Veerdonk et al. 2022).

To address these critical knowledge gaps, we recently elucidated molecular differences between and within two ARDS etiologies—COVID-19 and bacterial sepsis (Batra et al. 2022). Extending this blood-based ARDS comparison, we here performed a similar analysis on urine samples. It has been suggested that urine-based molecular profiles reflect an individual’s physiological changes (Wu and Gao 2015) and have the potential to be used as diagnostic and prognostic biomarkers (Berry et al. 2015; Aregger et al. 2014; Gisewhite et al. 2021; Currie et al. 2018). Previous urine-based COVID-19 studies have made substantial efforts to determine molecular markers distinguishing COVID-19 from healthy controls or less severe COVID-19 cases (Li et al. 2020a, 2021; Bi et al. 2022; Tian et al. 2020). However, a detailed comparison of the molecular differences between two ARDS groups has so far been missing.

In this study, we analyzed urine samples from 59 ARDS patients, with COVID-19 (n = 42) and bacterial sepsis diagnosis (n = 17). We followed a two-step analysis workflow to elucidate the differences between the two ARDS groups. In the first part, we compared metabolomic and proteomic profiles between the two groups to identify differentially abundant molecules. For a systematic cross-omics analysis of these molecules, we performed a data-driven network analysis. In the second part of the study, we compared the molecular heterogeneity within each ARDS group. To this end, we associated the omics measurements with clinical manifestations, including acute kidney injury (AKI) incidence, platelet counts, PaO2/FiO2, and mortality. For further exploration and reproducibility of our findings, we share all results, analysis scripts, and de-identified omics data.

## Methods

### Patient population

The cohort was derived from the Weill Cornell Biobank of Critical Illness (WC-BOCI) at Weill Cornell Medical College (WCMC)/ New York Presbyterian (NYP). The process for recruitment, data collection, and sample processing has been described previously (Finkelsztein 2017; Dolinay et al. 2012; Schenck 2019). Patients in the WC-BOCI database were admitted to the intensive care unit with valid consent between 2015 and 2020. Bacterial sepsis patients (n = 17) were recruited between June 2015 and January 2019 and COVID-19 patients were recruited between March 2020 and April 2020 (n = 42). Clinical data such as demographics, vital signs, labs, and ventilator parameters were obtained through the Weill Cornell-Critical Care Database for Advanced Research (WC-CEDAR) and the Weill Cornell Medicine COVID Institutional Data Repository (COVID-IDR). Additional clinical data were obtained through manual abstraction from the electronic health records.

This cohort included 47 (79.7%) males and 12 (20.3%) females, with a median age of 58.3. The overall mortality rate was 27.1%, with 10 out of 42 in COVID-19 ARDS and 6 out of 17 in bacterial sepsis-induced ARDS. 45.8% of patients suffered from acute kidney injury (AKI), with 15 out of 42 in COVID-19 ARDS and 12 out of 17 in bacterial sepsis-induced ARDS. The sequential organ failure assessment (SOFA) index was comparable between the two groups, with a median of 10 in the COVID-19 group and 9 in the bacterial sepsis group. Detailed demographics of the patient cohort are provided in Additional file 1: Table S1.

### Clinical manifestations

Below are the definitions used to diagnose the clinical manifestations used in this study.

#### Acute respiratory distress syndrome (ARDS)

ARDS was assessed using the Berlin definition (Ranieri et al. 2012), and followed by a review of the subject’s history, arterial blood gas, and chest X-ray by two independent pulmonary and critical care attendings to adjudicate the diagnosis. For bacterial sepsis-induced ARDS, an additional criterion was used as outlined in The Third International Consensus Definitions for Sepsis and Septic Shock (Singer et al. 2016). For diagnosis of COVID-19, a positive viral swab of the nasopharynx tested for SARS-CoV-2 via RT-PCR was required. Patients were classified as septic if they had a SOFA score ≥ 2, and had a clinically documented or suspected infection that upon final adjudication was deemed to be the source of organ dysfunction.

#### Acute kidney injury (AKI)

‘Kidney Disease: Improving Global Outcomes’ definition (KDIGO) was used to diagnose AKI. To this end, either of the following criteria was required: (a) serum creatinine change of greater than or equal to 0.3 mg/dL within 48 h, (b) serum creatinine greater than or equal to 1.5 times the baseline serum creatinine known or assumed to have occurred within the past 7 days, (c) urine output less than or equal to 0.5 mL/kg/h for 6 h (Khwaja 2012).

### Sample handling

Urine specimens were obtained from patients admitted to ICU at WCMC/NYP. Briefly, urine samples were centrifuged, and the supernatant was stored at − 80 °C until the omics profiling was performed. An electronic informed consent was obtained from all subjects for inclusion. For bacterial-sepsis ARDS, the median time of sample collection was 1.8 days after admission, with an interquartile range: 1.0–2.0, and for COVID-19 ARDS, the median was 7.6 days with an interquartile range: 3.5–9. Samples from both ARDS groups were profiled at the same time in the year 2020.

### Proteomic profiling

Proteomic profiling was performed by the Proteomics Core of Weill Cornell Medicine-Qatar using the Olink platform (Uppsala, Sweden) (Batra et al. 2022). Briefly, manufacturer's instructions were followed to profile the samples using four panels including Inflammation, Cardiovascular II, and Cardiovascular III panels. Thorough quality assurance/quality control (QA/QC) was performed to monitor the assay's incubation, extension, and detection steps. For (Ct) value extraction, Fluidigm’s reverse transcription-polymerase chain reaction (RT-PCR) analysis software was used at a quality threshold of 0.5 and linear baseline correction. Further processing of Ct values was performed using the Olink NPX manager software (Olink, Uppsala, Sweden).

### Metabolomic profiling

Metabolic profiling was performed by Metabolon, Inc (Morrisville, NC) using ultrahigh performance liquid chromatograph-tandem mass spectroscopy (UPLC-MS/MS) (Batra et al. 2022). Briefly, samples were subjected to methanol extraction and then divided into four aliquots for each of the mass spectroscopic methods. Rigorous quality assurance/quality control (QA/QC) was performed to monitor instrument performance and aid in chromatographic alignment. The four mass spectroscopic methods used were optimized for acidic positive ion hydrophilic compounds, acidic positive ion hydrophobic compounds, and basic negative ions, the fourth aliquot was analyzed via negative ionization. For metabolite identification, Metabolon’s proprietary software was used to deliver high-quality abundances of metabolites.

### Data processing

Metabolomic and proteomic profiles were preprocessed before downstream analysis: Molecules with more than 25% missing values were removed, leaving 708 out of 1112 metabolites and 266 out of 276 proteins. Probabilistic quotient normalization (Dieterle et al. 2006) was used to correct sample-wise variation in the data. Data was log_2_ transformed, followed by k-nearest-neighbor-based imputation (Do 2018) for the remaining missing values. Abundance levels of the following ten proteins were measured in duplicates by Olink panels and were therefore averaged: CCL3, CXCL1, FGF-21, FGF-23, IL-18, IL-6, MCP-1, OPG, SCF, and uPA. All data processing was performed using the maplet R package (Chetnik et al. 2022).

### Differential analysis of molecules

For association analysis, we used linear models with metabolites/proteins as the dependent variable and diagnosis/clinical manifestations as independent variables. Further factors such as age, sex, and BMI were not used as covariates in the models, since they are considered determinants of disease severity themselves (Docherty 2020). To control the false discovery rate, the Benjamini-Hochberg (BH) method (Benjamini and Hochberg 1995) was used to correct p-values. All analyses were performed using the maplet R package (Chetnik et al. 2022).

### Pathway annotation and filtering

For functional annotation of the differently abundant molecules, we used Metabolon’s ‘sub-pathway’ groups and signaling pathways from KEGG (Kanehisa et al. 2012) for metabolites and proteins, respectively. Additional file 2: Table S2 contains the complete list of annotations. For our analysis, we considered Metabolon’s sub-pathways with the term ‘metabolism’ and non-disease KEGG pathways with at least 3 significant molecules.

### Multi-omic network inference

To generate a multi-omic data-driven network we created a Gaussian graphical model (GGM) using the GeneNet R package (Schäfer and Strimmer 2005). GGMs are a partial correlation-based approach for identifying statistical connections among the molecules. To construct the network, pair of molecules (nodes) with significant partial correlations at 5% FDR were included and were connected with an edge. Following this, these nodes were annotated based on the statistical association results between the ARDS groups. To this end, a ${p}_{score}$ was computed using the following formula: ${p}_{score}=-{\mathrm{log}}_{10}\left(p.adj\right)\cdot d$, where $p.adj$ is the adjusted p-value of the association, and *d* is the direction (− 1/1) of the association based on test statistic (positive or negative association with the outcome). This score was used to color the nodes in the network.

## Results and discussion

### Molecular associations differentiating COVID-19 and bacterial sepsis-induced ARDS

**Fig. 1:**
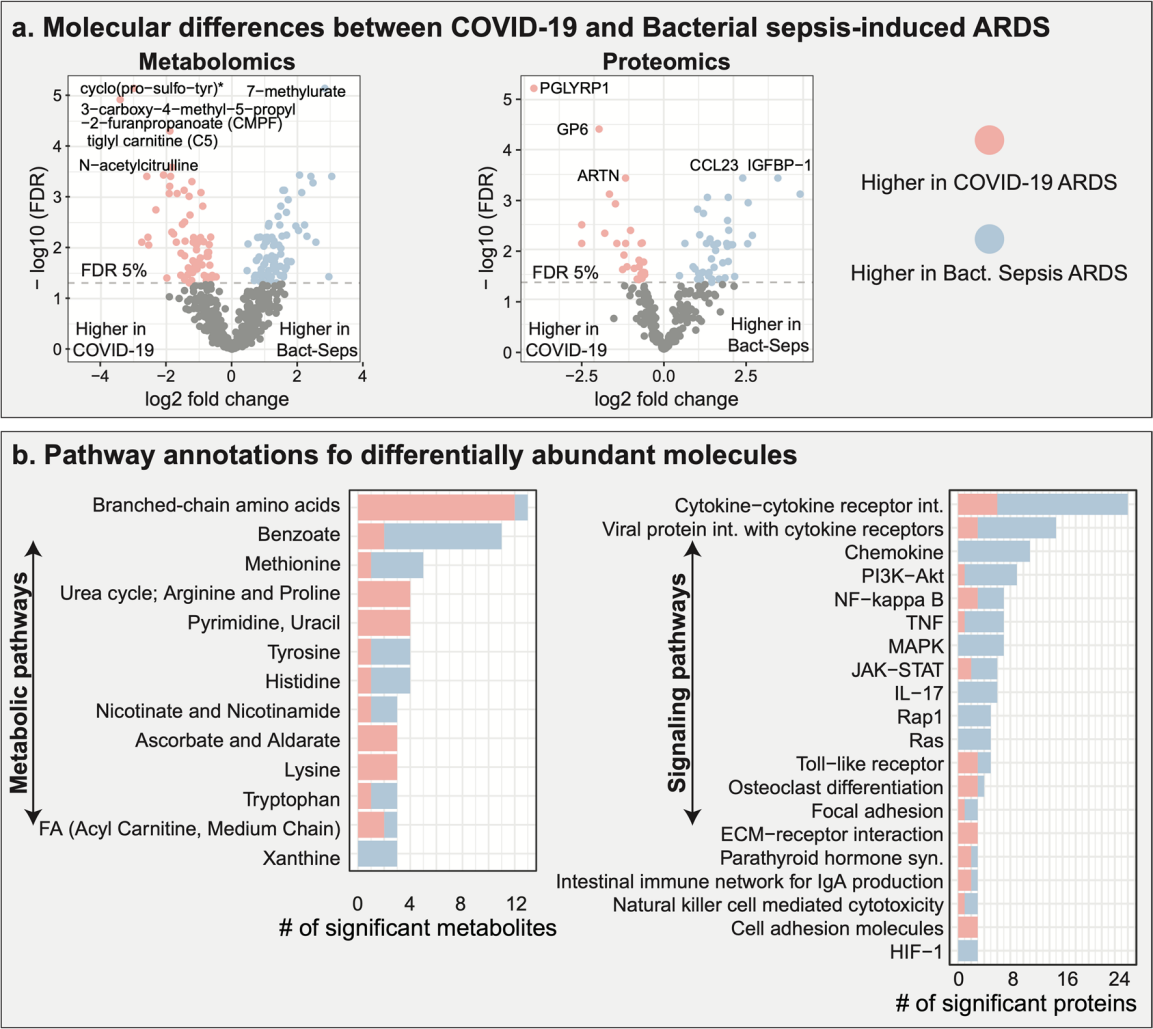
Molecular signature of COVID-19 ARDS compared to bacterial sepsis-induced ARDS.** a** Differentially abundant molecules (150 metabolites, 70 proteins) between the two ARDS groups. **b** Functional annotations of differentially abundant metabolites and proteins at the pathway level. Overall, 33 metabolic and signaling pathways with three or more significant molecules were deregulated between the two ARDS groups. FA: fatty acid

To identify the molecular differences between COVID-19 ARDS and bacterial sepsis-induced ARDS, urine-based metabolomic and proteomic profiles from 59 samples were analyzed (n = 42 COVID-19, and n = 17 bacterial sepsis). At a 5% false discovery rate (FDR), 220 molecules were significantly different between the two groups, representing 150 metabolites (70 higher in COVID-19 ARDS and 80 lower), and 70 proteins (28 higher in COVID-19 and 42 lower) (Fig. 1a). The results of this analysis are available in Additional file 2: Table S2. To aid the functional interpretation of these molecules, metabolites and proteins were annotated with ‘sub-pathway’ annotations provided by Metabolon and proteins were annotated with KEGG pathways (Kanehisa et al. 2012) (Additional file 3: Table S3). Top ranking pathways are shown in Fig. 1b. Two of the pathways we identified in this ARDS comparison, extracellular matrix (ECM) and cell adhesion molecules (CAMs), have also been implicated in previous urine-based studies comparing COVID-19 with a control group (Li et al. 2020b). In addition, blood-based studies have reported several of these pathways in the context of COVID-19 ARDS when compared to healthy controls, including amino acid metabolism, lipid metabolism, urea cycle, MAPK, PI3K-Akt, and JAK-STAT signaling (Hou et al. 2020; Grimes and Grimes 2020; Kalil et al. 2021; Guimarães et al. 2021; Montaldo et al. 2021). Taken together, we identified 220 molecules that were differentially abundant between the two ARDS groups, with 33 distinct biological pathways that had three or more significant molecules.

### ARDS-related interaction of mitochondrial dysfunction and ECM organization

**Fig. 2:**
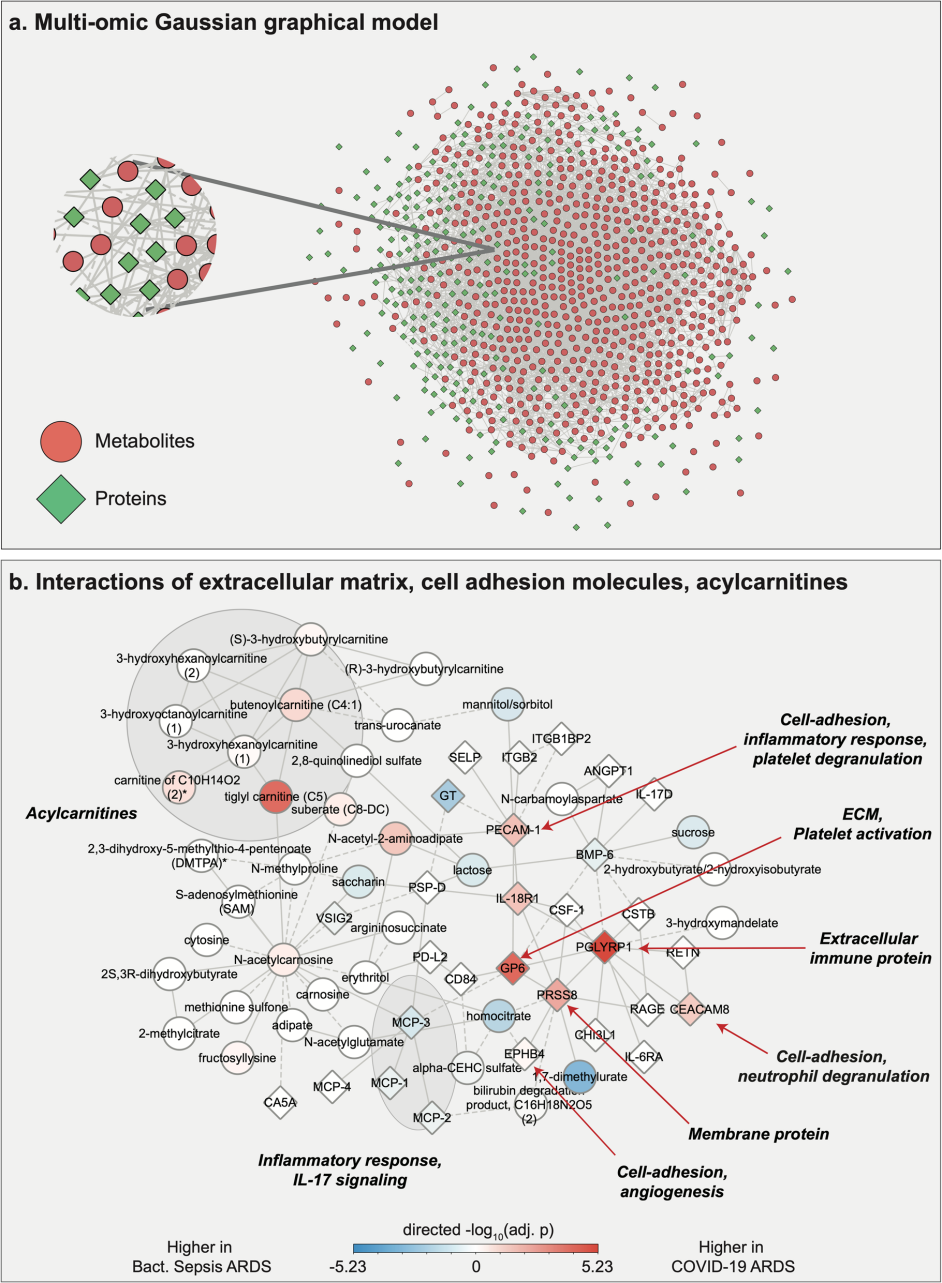
Multi-omic network and extracted ECM/CAM/acylcarnitine subnetwork.** a** Gaussian graphical model (GGM) of metabolites and proteins. Shapes and colors of the molecules in the network are based on the two omics types. **b** Subnetwork extracted from the full multi-omic GGM, built around tiglyl carnitine and GP6. The observed molecular interactions suggest an interplay of ECM derangement, inflammation, and mitochondrial dysfunction in ARDS pathogenesis

Predefined pathway annotations provide context for already well-characterized biological processes; however, the insights they provide into cross-omics associations are limited. Therefore, we generated a data-driven multi-omic interaction network based on Gaussian graphical models (GGM) (Schäfer and Strimmer 2005). In earlier studies, we have shown that partial correlation-based GGMs reconstruct valid biochemical interactions from omics data in an unbiased fashion and can even identify previously unknown interactions between molecules (Krumsiek et al. 2011; Do et al. 2017; Benedetti et al. 2017). The data-driven network contained 3566 statistically significant interactions between the 708 metabolites and 266 proteins (Fig. 2a). It was then annotated using the molecules that were differentially abundant between ARDS groups. An interactive version of the network is available in Additional file 6 for further exploration.

We then generated a subnetwork focusing on several processes that have previously been implicated in COVID-19, namely mitochondrial dysfunction (Ajaz et al. 2021), coagulopathy via cell-adhesion molecules (CAMs) and platelet activation (Tong et al. 2020; Sriram and Insel 2021). To this end, we chose two molecules belonging to these processes which were also among the top metabolomic and proteomic hits in Fig. 1a: Tiglyl carnitine, an acylcarnitine that represents mitochondrial function (Knottnerus et al. 2018), and glycoprotein 6 (GP6), which is involved in the extracellular matrix (ECM) and the platelet activation pathway.

The subnetwork was constructed by including tiglyl carnitine, GP6, and all of their first- and second-degree network neighbors, i.e., nodes that were separated from the two molecules by one or two edges in the network. The resulting subnetwork consisted of 66 molecules (37 metabolites, 29 proteins) with 106 interactions among them (Fig. 2b). Within this subnetwork, tiglyl carnitine and GP6 were connected via MCP-3 and *N*-acetylcarnosine. The neighborhood of tiglyl carnitine consisted of other acylcarnitines, including butenoylcarnitine (C4:1), (S)-3-hydroxybutyrylcarnitine, and 3-hydroxyhexanoylcarnitine, all of which were higher in COVID-19 compared to bacterial sepsis-induced ARDS. The neighborhood of GP6 consisted of additional proteins related to ECM or CAMs, including EPHB4, CECAM8, and PECAM1, all of which were higher in COVID-19 compared to bacterial sepsis-induced ARDS. The mediating inflammatory MCP-3 protein was connected to other MCP proteins, which were higher in bacterial sepsis-induced ARDS than in COVID-19.

Overall, within the subnetwork, we observed cross-omics connections between clusters of CAMs/ECM and a group of acylcarnitines, mediated by a group of inflammatory MCP proteins. We speculate that the underlying interplay of these depicted biological processes might play a role in ARDS pathogenesis.

### ARDS-specific heterogeneity of molecular associations across clinical manifestations

In the second part of our study, we tested ARDS group-specific molecular associations with four clinical manifestations: acute kidney injury (AKI), platelet count, patient’s oxygen in arterial blood to the fraction of the oxygen in the inspired air (PaO2/FIO2) ratio, and mortality. In the bacterial sepsis-induced ARDS group, no significant associations with any of these clinical manifestations were identified (5% FDR). In COVID-19 ARDS, there were 10 molecules associated with AKI, including 8 metabolites and 2 proteins, no molecules associated with platelet count, 6 metabolites associated with PaO2/FIO2, and 61 molecules associated with mortality, including 1 metabolite and 60 proteins. Thus, a molecular comparison of heterogeneous presentations across the two ARDS groups was not feasible. Detailed results are available in Additional file 4: Table S4 and Additional file 5: Table S5.

**Fig. 3:**
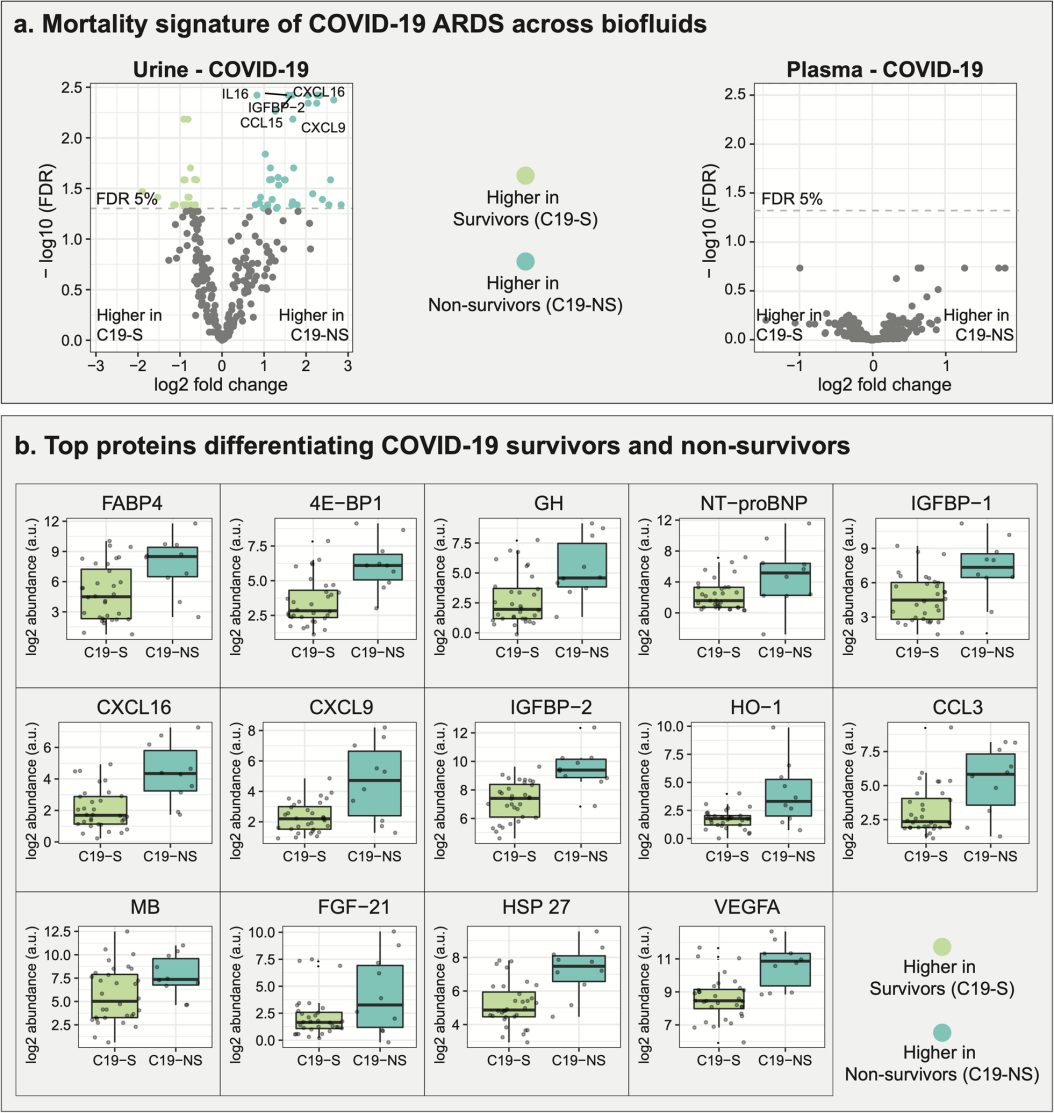
Proteomics-based mortality signature.** a** Differentially abundant proteins in COVID-19 survivors and non-survivors, as observed in two bodily fluids, urine (*n* = 42) and plasma (*n* = 43, one additional patient). 60 proteins were significant in urine proteomic profiles, while none of the proteins measured in the plasma of the same patients were associated with mortality. **b** Top 14 differential proteins from COVID-19 urine-based mortality signature with log2 fold change larger than or equal to 2 at 5% FDR

In the following, we focused on the proteomic mortality signature distinguishing survivors and non-survivors of COVID-19. Among 60 proteins that were significantly different between survivors and non-survivors, 22 were higher in survivors, 38 higher in non-survivors (Fig. 3a, left). Remarkably, in our recent plasma-based study (Batra et al. 2022), we did not find any proteins that were associated with mortality in the same COVID-19 patients (Fig. 3a, right). Of note, the plasma samples were taken from the same patients (including one additional patient), at the same time as the urine samples and were profiled using the same technology.

For further investigation of the urine-based COVID-19 mortality signature, we selected significant proteins with log2 fold changes larger than 2 (Fig. 3b). Interestingly, several of these 14 proteins have previously been described as biomarkers of pathologies that are linked to ARDS. For example, NT-proBNP in the urine of preterm infants has been shown to inform about pulmonary hypertension (Naeem et al. 2020). IGFBP-2 is an indicator of pulmonary arterial hypertension (PAH) (Yang et al. 2020) and can predict a decline of kidney function in type 2 diabetes (Narayanan et al. 2012). FABP4 has been implicated in proteinuria and has been discussed as a marker of kidney glomerular damage (Okazaki 2014). CXCL16 is considered a urinary marker of poor renal outcome in diabetic kidney disease (Lee et al. 2021). HO-1 is a candidate biomarker for oxidative damage in obstructive nephropathy (Li et al. 2012). VEGFA leads to increased inflammation in severe COVID-19 (Huang et al. 2019).

Taken together, these mortality-associated proteins have been implicated in ARDS-linked manifestations, including kidney dysfunction, pulmonary hypertension, and inflammation (Brault et al. 2020; Revercomb et al. 2020; Legrand et al. 2021). This provides insights into the potential pathophysiological processes behind the development of severe ARDS.

## Conclusion

In this study, we presented a first urine-based multi-omic comparison of COVID-19 ARDS and non-COVID-19 ARDS. We compared 42 COVID-19 ARDS patients to 17 bacterial sepsis-induced ARDS patients using untargeted metabolomics (708 metabolites) and targeted proteomics (266 proteins). There were two main findings from our work. First, the multi-omic network approach highlighted the interplay of mitochondrial dysfunction and ECM derangement in ARDS pathogenesis. Second, we identified a proteomics-based mortality signature in COVID-19 ARDS patients. Notably, within the bacterial sepsis-induced ARDS group, no metabolites or proteins were found to be associated with any of the four clinical manifestations tested. In the following paragraphs, we discuss the two novel findings from our study.

Our multi-omic network-based analysis indicated an ARDS-related link between CAMs/ECM and mitochondrial dysfunction represented by acylcarnitines. In the analyzed subnetwork, the connections between these different biological processes were mediated by inflammatory proteins. Previous COVID-19 studies have already individually implicated these processes in ARDS, but have not proposed a link between these pathways in the context of ARDS (Li et al. 2020b; Ajaz et al. 2021; Knottnerus et al. 2018). Moreover, mechanistically, ECM, CAMs, and acylcarnitines have individually been linked with inflammation (Sorokin 2010; González-Amaro et al. 1998; Rutkowsky et al. 2014). Our findings now highlight the potential synergy between these different cellular pathways in ARDS.

The proteomics-based mortality signature distinguishing COVID-19 survivors and non-survivors is another potentially novel finding from our study. Surprisingly, the mortality signal was absent in plasma proteomics profiles of the same patients. This could reflect frequent kidney involvement in severe COVID-19, which leads to the poor renal outcomes observed in COVID-19 patients (Legrand et al. 2021). Moreover, the signature contains several proteins implicated in pathological processes that have been linked to ARDS, including inflammation, kidney dysfunction, and pulmonary hypertension (Brault et al. 2020; Revercomb et al. 2020; Legrand et al. 2021). In terms of clinical stratification approaches, a higher-powered study will be needed to assess whether machine learning models based on our signature are able to predict mortality in COVID-19 ARDS patients.

We recognize that our study design has several limitations. (1) Since the patients of the two ARDS groups were collected several years apart, differences in sample collection and storage protocols may lead to unaccountable variation across measurements. (2) Our cohort has a limited sample size (n = 59), with imbalanced ARDS groups (42 COVID-19 versus 17 bacterial sepsis patients). This relatively small sample size could have led to false negatives in our analysis, especially within the bacterial sepsis group. (3) Since the coverage of the metabolomics and proteomics platforms is limited, there is potential for missed associations with unmeasured molecules. (4) Our study was limited to statistical associations in a single cohort since we did not have access to an independent cohort for replication.

In conclusion, we presented a first urine-based multi-omic analysis of COVID-19 ARDS compared to bacterial sepsis-induced ARDS. Our analysis shows molecular similarities and differences between the two ARDS groups. The most striking finding was a proteomics-based mortality signature specifically for COVID-19 ARDS, which will require further investigation as a potential early biomarker for mortality.

## Supplementary Information

**Additional file 1: Table S1.** Patient demographics and clinical manifestations.

**Additional file 2: Table S2.** Molecules differently abundant between COVID-19 ARDS and Bacterial sepsis-induced ARDS

**Additional file 3: Table S3.** Pathways associated with molecules differently abundant between COVID-19 ARDS and Bacterial sepsis-induced ARDS

**Additional file 4: Table S4.** Metabolites associated with clinical manifestations within COVID-19 ARDS and Bacterial sepsis-induced ARDS.

**Additional file 5: Table S5.** Proteins associated with clinical manifestations within COVID-19 ARDS and Bacterial sepsis-induced ARDS.

**Additional file 6.** **File s1.** Interactive multi-omic network from Figure 2.

## Data Availability

The preprocessed data used in this study can be downloaded at https://doi.org/10.6084/m9.figshare.20260998.v1; All R scripts to generate the tables and figures of this paper are available at https://github.com/krumsieklab/covid-ards-urine
